# Molecular and biochemical pathologies in human alcohol-related cerebellar white matter degeneration

**DOI:** 10.3389/adar.2025.15342

**Published:** 2025-11-03

**Authors:** Suzanne M. de la Monte, Ming Tong

**Affiliations:** ^1^ Departments of Pathology and Laboratory Medicine, Neurology, Neurosurgery, and Medicine, Rhode Island Hospital, Brown University Health, Alpert Medical School of Brown University, Providence, RI, United States; ^2^ Department of Medicine, Rhode Island Hospital, Brown University Health, Alpert Medical School of Brown University, Providence, RI, United States

**Keywords:** alcohol, human, white matter, oligodendrocytes, gliosis neurodegeneration, Notch

## Abstract

**Background:**

Alcohol use disorder (AUD) marked by heavy chronic or binge alcohol consumption, causes cerebellar and white matter (WM) atrophy with cognitive-motor impairments. Major pathological features of alcohol-related brain damage (ARBD) include alterations in WM integrity with myelin loss, and cerebellar degeneration with neuronal loss.

**Purpose:**

This study characterizes molecular and biochemical oligodendrocyte-related pathology in cerebellar tissue from donors with AUD to better understand the mechanisms of ARBD in humans.

**Methods:**

Cores of cerebellar vermis, including cortex and underlying WM from adult human postmortem AUD and control brains, were processed for RNA and protein analyses using duplex and multiplex panels.

**Results:**

AUD cerebellar WM had significant alterations in immature and mature oligodendrocyte protein and mRNA expression, and reduced expression of hepatocyte growth factor, Akt and GSK-3β signaling molecules, and Notch pathway activation. Moreover, the only significant AUD-related alteration in cerebellar cytokine/chemokine expression was reduced IL-16 immunoreactivity.

**Conclusion:**

Human AUD WM degeneration is associated with oligodendrocyte dysfunction, which mechanistically could be mediated by impairments in insulin/IGF signaling through Akt/GSK-3β or Notch pathway activation. Future studies should focus on the non-invasive detection and monitoring of AUD-related oligodendrocyte pathology through the analysis of cell-type-specific exosomes isolated from peripheral blood.

## Introduction

Alcohol related brain damage (ARBD) is the most significant consequence of alcohol use disorder (AUD), which is linked to chronic heavy or binge alcohol abuse [1]. ARBD-associated brain atrophy prominently targets the frontal lobes, temporal lobes, diencephalon, cerebellum, and white matter (WM) [2–5], the severities of which correlate with peak levels of daily and lifetime alcohol consumption [3, 6, 7]. Heavy drinking (over years) is the most common and preventable cause of cerebellar degeneration [8]. However, beyond the absolute levels of alcohol consumed, susceptibility to ARBD is influenced by cofactors such as smoking and its related tobacco nitrosamine exposure [9, 10], genetics, ethnicity, nutrient intake [11–16], thiamine deficiency, Wernicke’s encephalopathy [6, 17, 18], cannabis use [19], HIV infection [20], neuroinflammation [21, 22], and alcohol withdrawal-associated excitotoxic/metabolic injury [23–25].

Alcoholic cerebellar degeneration typically involves the anterior vermis [26, 27], adversely affecting both cortical and white matter structures. Alcohol’s neurotoxic and degenerative effects on cerebellar motor pathways impair coordination, visuospatial language skills, psychomotor speed, and emotional processing [28]. Additionally, the disruption of cerebellar-thalamic-cortical connections compromises cognitive and executive functions [29]. ARBD-related cerebellar cortical atrophy is marked by neuronal loss within the molecular and granule cell layers [2]. ARBD-associated cerebellar WM atrophy [3], which is pronounced in people with advanced liver disease [30], targets the cerebellar peduncles (major tracts) and the central and periventricular fibers [30], disrupting afferent and efferent projections that interconnect deep nuclei and the cortex, optic pathways, thalamus, and brainstem [31]. These adverse effects of chronic heavy alcohol consumption have been well-documented by neuroimaging [32, 33]. Furthermore, diffusion tensor imaging studies have suggested that disruption of WM micro-structural integrity [34, 35] is the underlying basis of AUD-associated WM atrophy. The pathologic correlates of the neuroimaging studies were revealed through histopathology and lipidomic mass spectrometry studies. In robust preclinical models, chronic heavy and binge alcohol consumption results in myelin loss (demyelination and impaired myelin maintenance) and axonal degeneration [36], which are associated with altered myelin lipid composition [37, 38]. Further studies showed that oligodendrocytes are important cellular targets of WM degeneration in ARBD [39–42].

To advance research on ARBD, it will be crucial to increase understanding of how all cell types are adversely affected by heavy alcohol consumption and the underlying mechanisms, which may vary among cell types. Previous preclinical and human brain studies showed that alcohol exerts its damaging effect on cerebellar cortical neurons, particularly granule cells, by inhibiting insulin and insulin-like growth factor (IGF) signaling pathways that support growth, survival, and energy metabolism [43–45], as well as through increased oxidative stress and free radical generation [46]. However, alcohol-related glial cell injury and degeneration have been less well studied. Our focus has been on alcohol’s targeting of oligodendrocytes in relation to WM degeneration. Oligodendrocytes synthesize integral membrane proteins, including myelin basic protein (MBP), myelin-associated glycoprotein (MAG), myelin oligodendrocyte glycoprotein (MOG), proteolipid protein (PLP) [47]. Preclinical studies have shown that alcohol exposure adversely impacts oligodendrocyte/myelin glycoprotein expression [48–51].

Experimental models have demonstrated that, like neurons, the underlying mediators of oligodendrocyte dysfunction in ARBD include impairments in insulin/IGF-1-Akt signaling [52, 53], as well as downstream pathways through the mechanistic target of rapamycin (mTOR) [41, 54], which is critical for oligodendrocyte myelin protein expression [52]. Further studies have demonstrated the importance of Notch signaling networks in regulating oligodendrocyte function [51, 52]. Additionally, during development, ethanol exposure has been shown to inhibit Notch pathways [53–55]. Mechanistically, ethanol’s inhibitory effects on Notch were found to be associated with reduced expression of aspartyl-asparaginyl-β-hydroxylase (ASPH) [54, 56, 57], which is regulated by insulin/IGF-Akt [58, 59]. ASPH plays a key role in Notch pathway activation via hydroxylation of its EGF-like domains, leading to cleavage and nuclear translocation of the Notch intracellular domain, which functions as a transcription factor for Hairy and enhancer of split (HES) and Hairy/enhancer-of-split related with YRPW motif (HEY) [60–62]. These concepts circle back to ARBD-mediated WM degeneration because recent experimental models demonstrated that ASPH is expressed in WM and that chronic ethanol exposure inhibits both ASPH and Notch [54]. Therefore, it is likely that the inhibitory effects of ethanol on insulin/IGF-Akt signaling impair ASPH’s crosstalk with Notch networks. Although this concept largely stems from developmental models, and similar studies have not yet been reported for adult/mature brains with ARBD, it is noteworthy that in adult humans with cerebral autosomal dominant arteriopathy with subcortical infarcts and leukoencephalopathy (CADASIL), Notch mutations cause WM atrophy and degeneration with myelin and oligodendrocyte loss [63]. Moreover, CADASIL WM degeneration is associated with impaired insulin/IGF-Akt signaling and ASPH expression [64].

In essence, alcoholic cerebellar degeneration is complex as both cortical and WM structures are adversely affected, and the consequences can broadly impact motor, cognitive, and behavioral functions. A key rationale for investigating the mechanisms of ARBD is to enhance diagnostic and treatment strategies. What’s reassuring is that cerebellar atrophy and degeneration can be partially reversed with abstinence [6, 65, 66]. Therefore, the goals of future investigations should include identifying disease stages and abnormalities that could potentially be addressed to either prevent or reduce the severity of ARBD, including cerebellar degeneration. The present study was designed to characterize the molecular and biochemical abnormalities in human cerebellar ARBD to determine the degree to which those pathologies resemble the findings in experimental models, which could then be better utilized to develop novel diagnostic and therapeutic strategies. To these ends, we utilized human postmortem cerebellar tissue to characterize the effects of AUD on oligodendrocyte/myelin function, insulin/IGF signaling, and ASPH-Notch networks.

## Methods

### Human subjects

Postmortem brain tissue samples were obtained from the New South Wales Brain Tissue Research Centre (BTRC) in Sydney, Australia. The NSW BTRC and its associated donor program have ethics approval from the NSW Government Health authority/University of Sydney to bank postmortem brains from deceased subjects with documented histories of alcohol abuse or normal controls (ref# X11-0107&HREC/11/RPAH/147). Under Australian law, people aged 18 or older are considered adults. The NSW BTRC has ethics approval that allows any adult to consent to brain donation. The present study includes cases between 40 years and 70 years of age. Prospective donors were provided with written and verbal information about the nature of the study, the procedures, and the evaluations involved. Prospective participants were screened for eligibility. Once they or their next of kin (in cases where consent was obtained via the coronial office) understood the nature and purpose of the study and what was being requested of them, they were asked to read and sign a written informed consent form agreeing to all aspects of the study. In essence, written informed consent was always obtained prior to entry into the study. All study data are kept confidential, and no information is revealed to any other sources. Incentives and compensations are not used for enrollment or continued participation. The research herein was conducted in accordance with the rules and regulations of the Institutional Review Boards at Brown University Health and Rhode Island Hospital in Providence, RI (USA), and at the University of Sydney, NSW, Australia. Permission for the investigators at Brown University Health to use the postmortem human tissue was obtained from the NSW Brain Bank, which includes the NSW BTRC. The research was performed with banked human brain tissue as part of an ongoing collaborative project with the University of Sydney. The use of deidentified human postmortem tissue for research meets Exemption Criteria 4 under 45CFR Part 46. The samples were de-identified prior to transfer from the BTRC to Brown University Health. A Tissue Transfer Agreement, outlining the conditions of tissue usage, was required to be completed prior to making the tissue samples available.

This human tissue research was approved by the BTRC Scientific Advisory Committee, the University of Sydney Human Research Ethics Committee (2018/HE000477), and the Brown University Health Institutional Review Board (CMTT/PROJ:#013024). All donors were free of other substance use disorders, and none of the participants had been enrolled in clinical trials. Relevant aggregate demographic, clinical, and postmortem data are provided in Table 1. The AUD and control groups each included 6 male subjects with similar mean ages (Years ± S.D.) (AUD: 57.33 ± 7.37; Control 57.83 ± 6.68) and age ranges (AUD: 50–70; Control: 50–69). Despite the similar number of years (±S.D.) of alcohol consumption (AUD: 34.0 ± 7.11; Control: 26.2 ± 4.27), the lifetime quantity (Kg) of alcohol consumed was significantly greater in the AUD (2,784 ± 1,465) than in the control (42.3 ± 35.5) group (p < 0.0001). Smoking was the only noted cofactor. Four of 6 AUD and 3 of 6 controls had smoking histories. The postmortem AUD mean brain weight (1,335 ± 133 g) was significantly lower than control (1,505 ± 115) (p = 0.02). The mean postmortem intervals to autopsy and brain pHs were similar for the two groups. Cores (6-mm diameter) of fresh frozen anterior cerebellar vermis were stored at −80 °C for later micro-dissection and processing for molecular and biochemical studies [6, 67].

**TABLE 1:** Human subjects.

Characteristics	AUD	Controls	P-value
# Cases	6	6	N.S.
Age (Years)	57.33 ± 7.37	57.83 ± 6.68	N.S.
Age (Range-Years)	50–70	50–69	N.S.
Male/Female (#)	6M/0F	6M/0F	N.S.
Drinking History (Years)	34.0 ± 7.11	26.2 ± 4.27	N.S.
Lifetime Alcohol (Kg)	2,784 ± 1,465	42.3 ± 35.5	<0.0001
Smoking History (Y/N)	4/6	3/6	N.S.
Postmortem Interval (Hours)	32.17 ± 24.81	22.17 ± 6.43	N.S.
Brain pH	6.66 ± 0.23	6.61 ± 0.21	N.S.
Brain Weight (g)	1,335 ± 133	1,505 ± 115	0.02

Characteristics of alcohol use disorder (AUD) and control deceased donors. Data correspond to either counts (#) or mean ± S.D. Intergroup comparisons of mean values were made using Student t-tests, and the proportions of males and females, as well as smokers, were compared using Chi-square tests. N.S. = not statistically significant.

### Tissue homogenization

Using a TissueLyser II instrument (Qiagen, Germantown, MD, USA) and 5-mm diameter stainless steel beads, two sets of fresh frozen brain tissue samples (100 mg each) from the anterior vermis were homogenized in 5 volumes of weak lysis buffer (50 mM Tris (pH 7.5), 150 mM NaCl, 5 mM EDTA (pH 8.0), 50 mM NaF, and 0.1% Triton X-100) or Radioimmunoprecipitation Assay (RIPA) buffer (20 mM Tris-HCl, pH 7.5, 150 mM NaCl, 1 mM EDTA, 1 mM EGTA, 1% NP-40, 1% sodium deoxycholate). The lysis buffers were supplemented with protease (1 mM PMSF, 0.1 mM TPCK, 2 μg/mL aprotinin, 2 μg/mL pepstatin A, 1 μg/mL leupeptin, 1 mM NaF, 1 mM Na_4_P_2_O_7_) and phosphatase (10 mM Na_3_VO_4_) inhibitors. The supernatants generated by centrifuging the samples at 14,000 rpm for 10 min at 4 °C were aliquoted and stored at −80 °C for later immunoassays. Protein concentrations were determined using the bicinchoninic acid (BCA) assay.

### Duplex enzyme-linked immunoassays (ELISAs)

Immunoreactivity to oligodendrocyte-glial proteins was assessed using duplex ELISAs [54]. The glial protein analyses were clustered into groups corresponding to immature oligodendrocyte/myelin proteins: 2′,3′-cyclic nucleotide 3′ phosphodiesterase (CNPase), proteolipid protein 1 (PLP), Platelet-derived growth factor receptor, alpha peptide (PDGFRA), and Group-specific component Vitamin D Binding (GALC); mature oligodendrocyte/myelin proteins: myelin-associated glycoprotein 1 (MAG), myelin oligodendrocyte glycoprotein (MOG), and myelin basic protein (MBP); and astrocyte proteins: nestin, vimentin, and glial fibrillary acidic protein (GFAP). Supplementary Table S1 lists the antibody sources, concentrations used, research resource identifier (RRID) numbers, and commercial validation methods. The duplex ELISA results were normalized to large acidic ribosomal protein (RPLPO) as the loading control because RPLPO immunoreactivity increases linearly with protein content between 10 ng and 80 ng/well [68]. To perform the duplex ELISAs, triplicate 50 ng protein samples, each in 50 µL bicarbonate binding buffer, were robotically distributed (EpMotion 330) into 96-well MaxiSorp plates. After overnight adsorption at 4 °C, non-specific binding sites were masked with Superblock TBS, and then the samples were incubated with primary antibodies (0.2–5.0 μg/mL) overnight at 4 °C. Immunoreactivity was detected with horseradish peroxidase (HRP)-conjugated secondary antibodies and the Amplex UltraRed soluble fluorophore. Fluorescence intensity was measured (Ex 530 nm/Em 590 nm) in a Spectra-Max M5 Multimode Plate Reader (Molecular Devices, Sunnyvale, CA, USA). After rinsing in Tris-buffered saline (TBS), the samples were incubated with biotin-conjugated anti-RPLPO, followed by streptavidin-conjugated alkaline phosphatase, and RPLPO immunoreactivity was detected with 4-Methylumbelliferyl phosphate (4-MUP) (Ex 360 nm/Em 450 nm). Fluorescence was measured in a SpectraMax M5. The calculated ratios of target protein to RPLPO were used for statistical comparisons.

### Multiplex ELISAs

Neuroinflammatory/metabolic effects in the brain were assessed using commercial magnetic bead-based cytokine/chemokine 11-Plex plus 10-Plex panels (BioRad; Supplementary Table S2). Akt pathway signaling was assessed using 7-Plex total and phosphoprotein commercial magnetic bead-based ELISAs (Supplementary Table S3). The assays were performed according to the manufacturers’ protocols. In brief, after incubating the samples with antibody-coated beads to capture the antigens, immunoreactivity detected with biotinylated secondary antibodies and phycoerythrin-conjugated streptavidin was measured in a Luminex MAGPIX instrument (Diasorin, Austin TX USA) with xPONENT software. MAGPIX calibration and verification standards were used throughout, and standard curves were generated for each analyte.

### Quantigene 2.0 RNA multiplex assay

Total RNA was extracted from fresh frozen tissue using QIAzol Lysis Reagent (Qiagen, Germantown, MD USA). The samples were analyzed for mRNA expression using two custom Quantigene 2.0 Multiplex panels. The 10-Plex Human Glial panel (QuantiGene 2.0 Plex Set Cat# 312185) was used to measure 2′,3′-cyclic nucleotide 3′ phosphodiesterase (*CNP*), Chondroitin Sulfate Proteoglycan 4 (*CSPG4*), *GFAP*, Kallikrein-related peptidase 6 (*KLK6*), *KLK8*, *MBP, MOG, PLP1*, glyceraldehyde-3-phosphate dehydrogenase (*GAPDH*), Hypoxanthine phosphoribosyltransferase 1 (*HPRT1*), and Ribosomal Protein L13a (*RPL13A*) mRNA expression (Supplementary Table S4). The 20-plex assay for human Insulin/Notch pathways (Affymetrix Inc., Santa Clara, CA USA, Cat# 312177) measured mRNA transcripts corresponding to insulin (INS), insulin-like growth factor 1 (*IGF1*), *IGF2*, insulin receptor (*INSR*), *IGF1R, IGF2R*, insulin receptor substrate 1 (*IRS1*), *IRS2, IRS4, ASPH, NOTCH1*, Jagged 1 (*JAG1*), Hairy and enhancer of split-1 (*HES1*), HES-related family bHLH transcription factor (*HEY1*), and hypoxia-inducible factor 1-alpha (*HIF1α*) (Supplementary Table S5). Quantigene 2.0 Multiplex (QGP) Assays permit direct mRNA quantification using xMAP Luminex beads and reporter signal amplification. RPL13a served as the internal control gene for normalizing the results. Cooperative hybridization and quantification were performed following the manufacturer’s protocol.

In brief, capture beads suspended in lysis buffer, blocking reagent, and an RNA probe set were distributed in 96-well plates. Total RNA (1 µg) was incubated with the reaction mixtures overnight with the xMAP fluorescent beads. Sterile nuclease-free water was used as the negative control. The samples were first incubated with a set of oligonucleotide probes (pre-amplifier, amplifier, and biotin-label), followed by streptavidin-conjugated R-Phycoerythrin (SAPE). The resulting fluorescent signals were detected with a Luminex MAGPIX instrument (Diasorin, Austin TX USA). MAGPIX calibration and verification standards were used throughout, ensuring the levels of SAPE fluorescence were proportional to RNA transcript abundance captured by the beads. After subtracting the probe-related background from the target median fluorescence intensity (MFI), the results were normalized to RPL13a.

### Data analysis

Biochemical and molecular assays were performed using two separate tissue cores per case, with all assays performed in triplicate and under code. Each set of triplicate data points per sample was averaged to generate a single value per case. The results from 6 AUD and 6 control samples were used to generate graphs and calculate the between-group differences in mean protein or mRNA expression with GraphPad Prism 10.4 software (GraphPad Software Inc., Boston, MA). Two-way/Mixed Model Analysis of Variance (ANOVA) tests with *post hoc* Šídák’s multiple comparison tests were used for data analysis. Data for the individual assays passed the D’Agostino & Pearson test for normality (alpha = 0.05). Violin plots display the distribution of results, including the median, first and third quartiles, and upper and lower data points. Additionally, between-group comparisons were made using heatmaps to compare the effects of AUD within the glial, inflammatory, trophic factor, Akt pathway signaling protein biomarker panels, and glial, Insulin pathway, and Notch pathway mRNA biomarker panels. Software-generated statistically significant (p ≤ 0.05) between-group differences are displayed in the Tables and Graphs.

### Materials and instruments

The ELISA MaxiSorp 96-well plates, Bicinchoninic acid (BCA) reagents, horseradish peroxidase (HRP)-conjugated secondary antibodies, and Superblock (TBS) were purchased from Thermo-Fisher Scientific (Bedford, MA USA). The soluble fluorophores, Amplex UltraRed and 4-Methylumbelliferyl phosphate (4-MUP) were from Life Technologies (Carlsbad, CA, USA). Vector Laboratories Inc. (Newark, CA, USA) was the source of the Proton Biotin Protein Labeling Kit and Alkaline Phosphatase-conjugated Streptavidin. The 11-Plex+10-Plex MILLIPLEX MAP Human Cytokine Magnetic Bead Panels were purchased from Millipore (Burlington, MA, USA). The human Total 7-plex and Phospho-7-plex Akt Pathway kits and reagents were from ThermoFisher/Invitrogen (Camarillo, CA, USA). Vector Laboratories Inc. (Newark, CA, USA) was the source of the Protein Biotin Protein Labeling Kit and Alkaline Phosphatase-conjugated Streptavidin. All other fine reagents were purchased from CalBiochem/Millipore Sigma (Burlington, MA, USA), Pierce Chemical (Dallas, TX, USA), or Sigma-Aldrich Co. (St. Louis, MO, USA).

## Results

### Oligodendrocyte/glial protein expression

**FIGURE 1:**
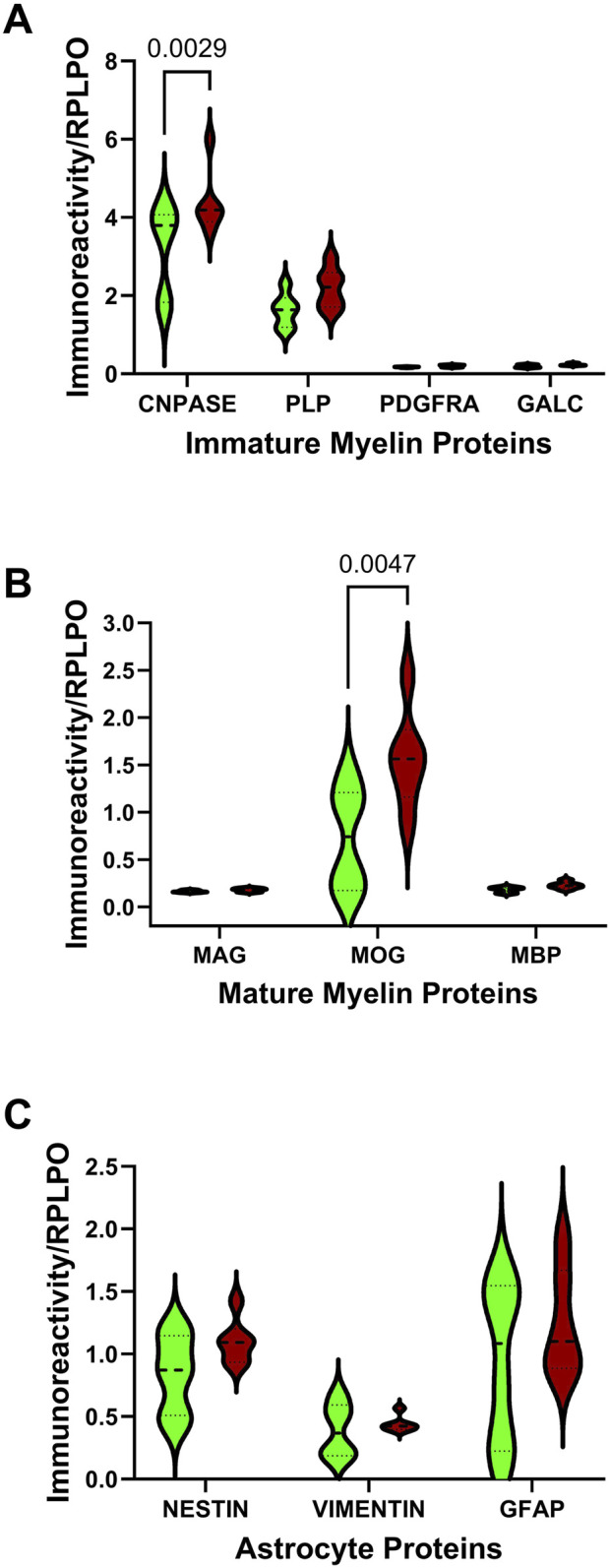
Effects of AUD on glial protein immunoreactivity in cerebellar tissue. Violin plots (median, quartiles, and range) display the control (green) versus AUD (brown) group differences in the levels of immunoreactivity corresponding to **(A)** immature myelin proteins (CNPase, PLP, PDGFRA, GALC), **(B)** mature myelin proteins (MAG, MOG, MBP), and **(C)** astrocyte markers (nestin, vimentin, GFAP) measured by duplex ELISA with results normalized to RPLPO. The units of measurement were fluorescent light units (FLU)/50 ng protein/RPLPO FLU. Data were analyzed by two-way ANOVA (Table 2) and *post hoc* multiple comparisons tests. Significant (p ≤ 0.05) differences are shown within the panels. See Supplementary Table S1 for abbreviation definitions and antibody information.

Duplex ELISAs measured immature (CNPase, PLP, PDGFRA, GALC) and mature (MAG, MOG, MBP) myelin oligodendrocyte proteins, and astrocyte proteins (Nestin, vimentin, GFAP). Immunoreactivity was normalized to RPLPO. Comparative effects of ethanol exposure on glial protein expression within each cluster were analyzed with two-way ANOVA tests. The analyses revealed significant AUD and glial biomarker effects on immature and mature myelin/oligodendrocyte proteins (Table 2). Additionally, significant glial biomarker effects were observed with respect to astrocytic proteins. Post-hoc multiple comparisons tests revealed significantly higher levels of CNPase (Figure 1A) and MOG (Figure 1B) in AUD relative to control cerebellar tissue. There were no significant effects of AUD on PLP, PDGFRA, GALC, MAG, MBP, Nestin, Vimentin, or GFAP immunoreactivities (Figures 1A–C).

**TABLE 2:** Cerebellum-two-way ANOVA ELISA results.

Biomarker type	AUD-factor F-ratio	p-Value	Biomarker F-ratio	p-Value	AUD x biomarker F-ratio	p-Value
Oligodendrocyte/Myelin Glial Biomarkers						
Immature Myelin	**7.954**	**0.0074**	**116.5**	**<0.0001**	*2.817*	*0.0512*
Mature Myelin	**8.432**	**0.0069**	**38.91**	**<0.0001**	**6.864**	**0.0035**
Astrocyte	2.789	0.1053	**12.26**	**0.0001**	0.4018	N.S.
Inflammatory Biomarkers:Cytokines/Chemokines						
All Inflammatory Factors	**4.594**	**0.0328**	**85.74**	**<0.0001**	**2.742**	**0.0008**
Proinflammatory Cytokines	*2.941*	*0.0885*	**85.38**	**<0.0001**	**2.749**	**0.0147**
Proinflammatory Chemokines	2.112	N.S.	**30.23**	**<0.0001**	**3.135**	**0.0106**
Anti-inflammatory	1.356	N.S.	**265.8**	**<0.0001**	0.1507	N.S.
Trophic Factor Biomarkers						
Growth Factors	**8.527**	**0.0041**	**857.1**	**<0.0001**	**3.728**	**0.0035**
Akt Pathway Biomarkers						
Total Proteins	**7.461**	**0.008**	**38.05**	**<0.0001**	**2.824**	**0.016**
Phosphoproteins	1.863	N.S.	**22.41**	**<0.0001**	1.387	N.S.
P/T Relative Phosphorylation	**5.253**	**0.025**	**75.84**	**<0.0001**	1.075	N.S.

Cerebellar tissue samples from human alcohol use disorder (AUD) or control deceased participants were analyzed by duplex or multiplex ELISAs (see *Methods* and Supplementary Tables S1–S3). Two-way ANOVA tests compared the effects of AUD, clustered biomarkers, and AUD x biomarker interactions. The calculated F-ratios and p-values corresponding to the ANOVA results are indicated. Significant effects (p ≤ 0.05) are highlighted with bold font. N.S. = not significant. The results of *post hoc* Šídák’s multiple comparison tests are shown in Figures 1, 4, 5, 7.

### Oligodendrocyte/glial mRNA expression

**FIGURE 2:**
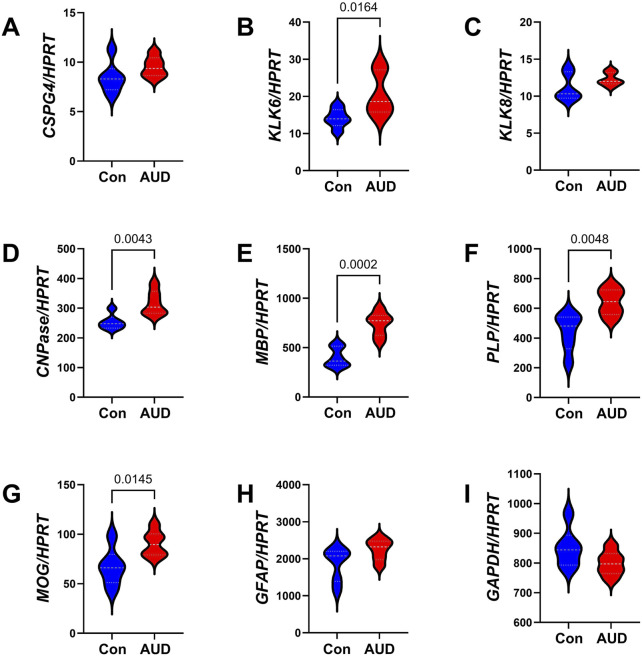
Effects of AUD on glial mRNA expression in cerebellar tissue. Cerebellar tissue mRNA transcripts were measured using a custom multiplex magnetic bead-based RNA hybridization assay with 1 µg purified total RNA per sample (see Methods). Violin plots (median, quartiles, and range) display between-group differences in the levels of gene expression corresponding to **(A)** CSPG4, **(B)** KLK6, **(C)** KLK8, **(D)** CNPASE, **(E)** MBP, **(F)** PLP, **(G)** MOG, **(H)** GFAP, **(I)** GAPDH, with results normalized to HPRT. Data were analyzed by two-way ANOVA (Table 3) and *post hoc* multiple comparisons tests. Significant (p ≤ 0.05) differences are shown within the panels. See Supplementary Table S4 for abbreviation definitions and full gene names.

**FIGURE 3:**
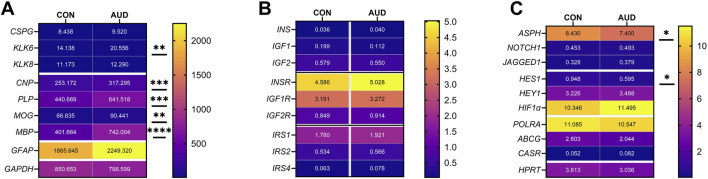
Gene expression heatmaps reflecting AUD effects on mRNA transcripts corresponding to **(A)** glial, **(B)** insulin/IGF pathway, and **(C)** Notch pathway genes. mRNA transcript abundances were measured with custom multiplex magnetic bead-based RNA hybridization assays. The results were normalized to *HPRT* or *RPL13a* (see Methods). Data were analyzed by two-way ANOVA (Table 3) and *post hoc* multiple comparisons tests (*p ≤ 0.05; **p < 0.01; ***p < 0.001; ****p < 0.0001). See Supplementary Tables S4, S5 for abbreviation definitions and full gene names.

A commercial custom multiplex RNA hybridization assay measured immature (*CSPG4, KLK6, KLK8,* and *CNPase*) and mature (*MBP, PLP,* and *MOG*) oligodendrocyte/myelin mRNA transcripts, *GFAP,* and *GAPDH,* with results normalized to the HPRT housekeeping gene. Two-way ANOVA demonstrated significant AUD, glial biomarker, and AUD x glial biomarker interactive effects on glial mRNA expression (Table 3). The *post hoc* multiple comparison tests detected significant AUD-related increases in *KLK6* (Figure 2B), *CNPase* (Figure 2D), *MBP* (Figure 2E), *PLP* (Figure 2F), and *MOG* (Figure 2G). In contrast, there were no significant effects of AUD on *CSPG4* (Figure 2A), *KLK8* (Figure 2C), *GFAP* (Figure 2H), or *GAPDH* (Figure 2I), an insulin-responsive gene [69]. The corresponding heatmap concisely depicts the broadly upregulated expression of immature and mature oligodendrocyte/myelin genes (Figure 3A).

**TABLE 3:** Cerebellum two-way ANOVA multiplex mRNA results.

mRNA Panel	AUD-Factor F-Ratio	p-Value	mRNA F-Ratio	p-Value	AUD x mRNA F-Ratio	p-Value
Glial Genes	**16.63**	**<0.0001**	**276.7**	**<0.0001**	**4.113**	**0.0003**
Insulin-IGF-IRS	0.842	N.S.	**188.2**	**<0.0001**	0.398	N.S.
Notch Pathway	0.977	N.S.	**307.7**	**<0.0001**	1.519	N.S.

Postmortem cerebellar tissue samples from human AUD or control donors were analyzed with custom Quantigene 2.0 Panels to measure mRNA expression of Glial, Insulin-IGF-IRS, and Notch Pathway Genes relative to HPRT or RPL13a as control (see Methods and Supplementary Tables S4, S5). The results were analyzed by two-way ANOVA comparing the effects of AUD, mRNA biomarker, and AUD x mRNA biomarker interactions. Calculated F-ratios and p-values corresponding to the ANOVA results are indicated. Significant effects (p ≤ 0.05) are highlighted with bold font. N.S. = not significant. The results of *post hoc* multiple comparisons tests are shown in Figures 2, 3, 6, 8.

### Inflammatory factor expression

**FIGURE 4:**
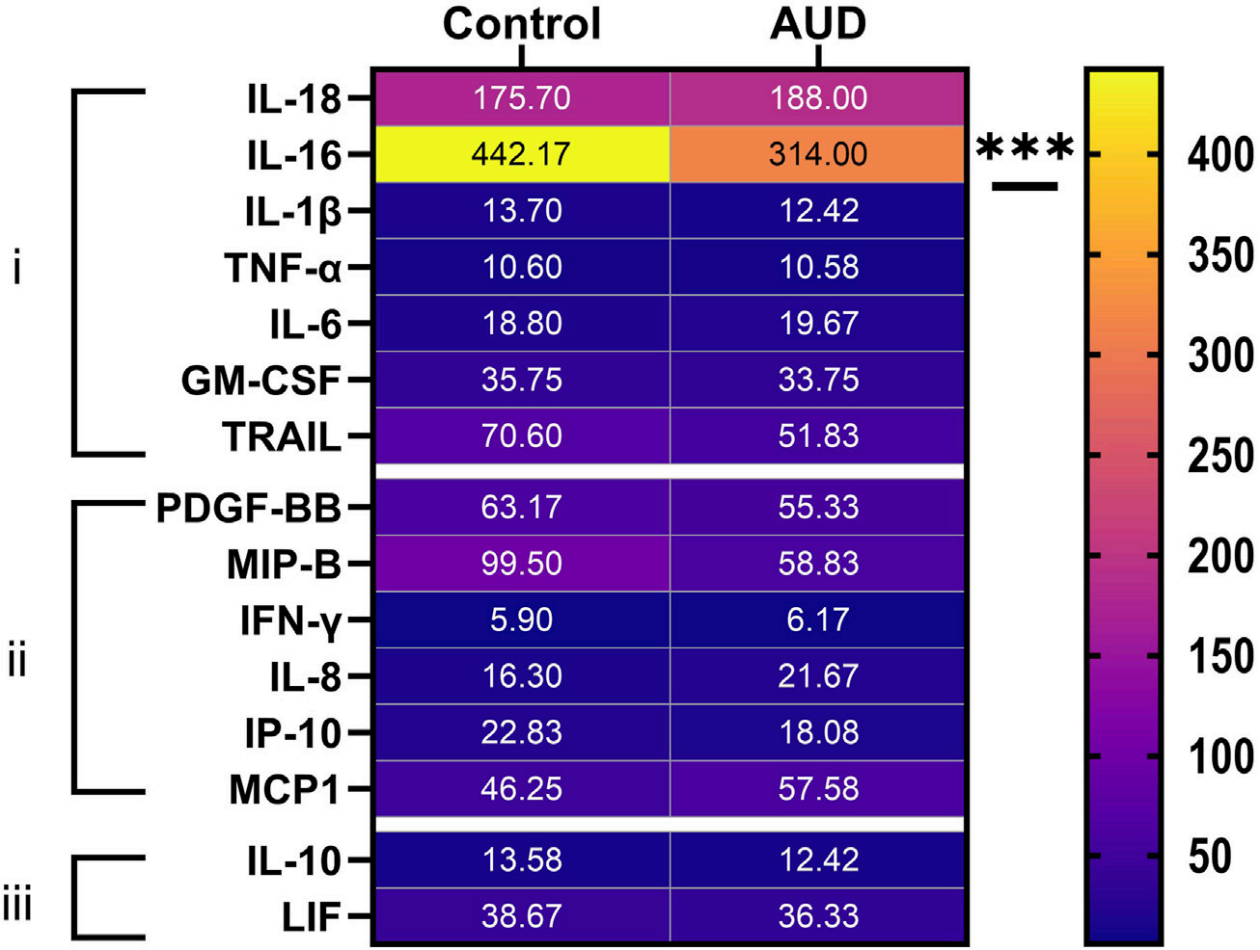
Heatmap display of (i) proinflammatory cytokine, (ii) proinflammatory chemokine, and (iii) anti-inflammatory cytokine expression in human control and AUD cerebellar tissue. The cytokine/chemokine molecules were measured in tissue homogenates using commercial magnetic bead-based multiplex ELISAs. The immunoreactivity results displayed correspond to pg/200 µg protein included in each assay. The data were analyzed by two-way ANOVA (see Table 2) with *post hoc* multiple comparison tests. The only notable between-group differences were a significant reduction in IL-16 in AUD. See Supplementary Table S2 for abbreviation definitions.

Multiplex ELISAs were used to measure proinflammatory cytokines, proinflammatory chemokines, and anti-inflammatory cytokines in cerebellar tissue homogenates. The objective was to assess the role of persistent inflammation as a mediator of ARBD. Two-way ANOVA detected significant inflammatory biomarker and AUD x inflammatory biomarker interactive effects on proinflammatory cytokines. Significant inflammatory biomarker and AUD x inflammatory biomarker interactive effects were observed for pro-inflammatory chemokines, and a significant inflammatory biomarker effect was observed with respect to anti-inflammatory cytokines (Table 2). The aggregate results, displayed as a heatmap (Figure 4), reveal that most inflammatory biomarkers were expressed at low levels in the cerebellar tissue. Among the 21 factors, only one significant between-group difference was detected, namely reduced levels of IL-16 (a pro-inflammatory cytokine) in AUD versus control cerebellar tissue. It is noteworthy that IL-16 was the most abundantly expressed of the inflammatory factors. Next in abundance was IL-18, which was similarly expressed in AUD and control samples. Re-analysis of log-transformed data to adjust for large differences in mRNA expression also failed to detect significant inter-group differences beyond IL-16. In essence, the other inflammatory mediators were either similarly expressed in AUD and control samples, or they had relatively high within-group variances, resulting in considerable overlap between groups.

### Trophic factors

**FIGURE 5:**
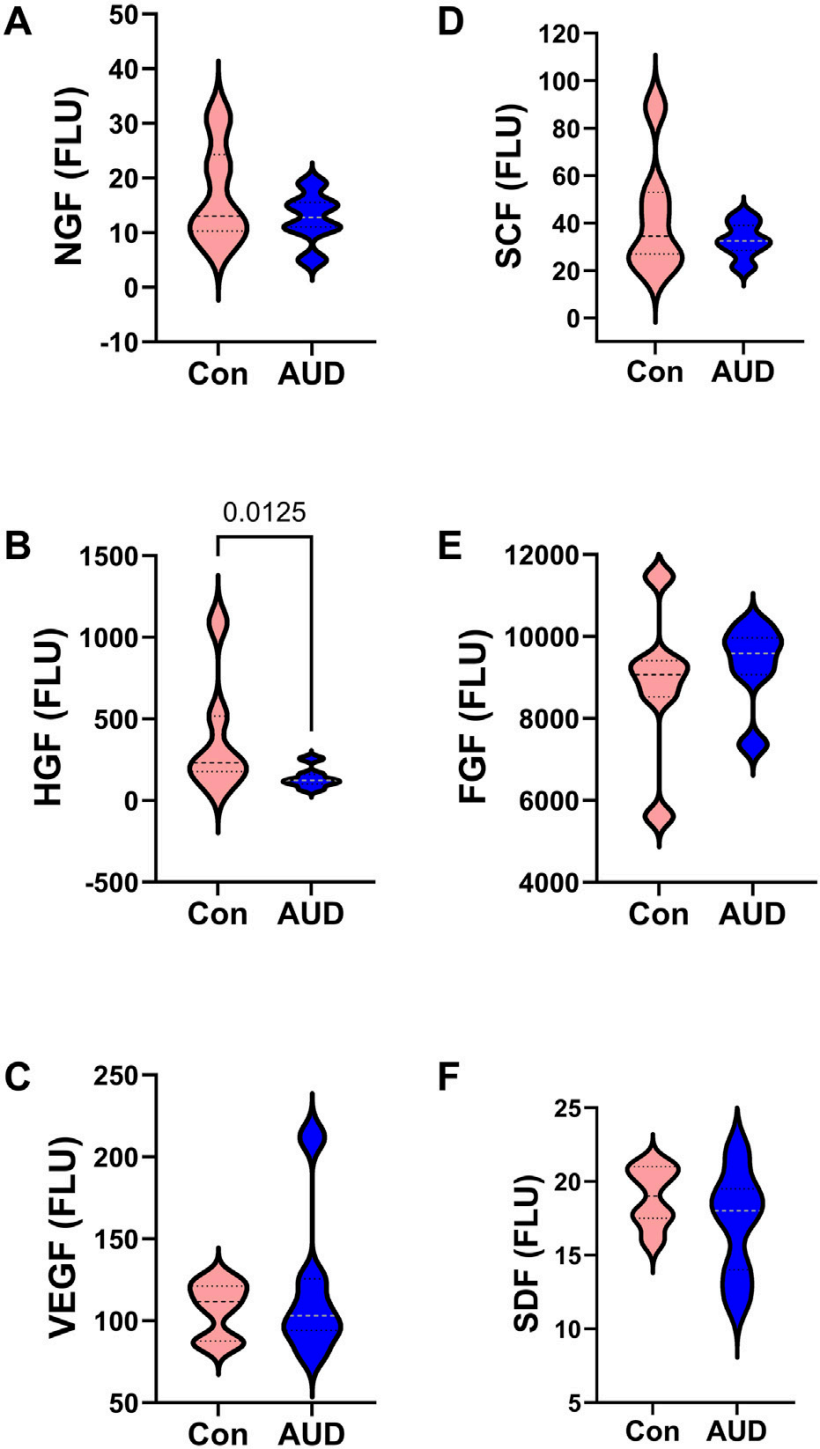
AUD alters cerebellar trophic factor immunoreactivity. Violin plots (median, quartiles, and range) display between-group differences in the levels of **(A)** NGF, **(B)** HGF, **(C)** VEGF, **(D)** SCF, **(E)** b-FGF, and **(F)** SDF measured with a magnetic bead-based multiplex ELISA. The immunoreactivity results displayed correspond to pg/200 µg protein included in each assay. Data were analyzed by two-way ANOVA (Table 2) and *post hoc* multiple comparisons tests. Significant (p ≤ 0.05) differences are shown within the panels. See Supplementary Table S2 for abbreviation definitions.

The trophic factors measured by multiplex ELISA included NGF, HGF, VEGF, SCF, FGF, and SDF (Figure 5). Two-way ANOVA tests demonstrated significant effects of AUD, trophic biomarker, and AUD x trophic biomarker interactive effects on trophic factor expression (Table 2). Post hoc multiple comparison tests demonstrated a significant inhibitory effect of AUD on HGF (Figure 5B), but not on NGF (Figure 5A), VEGF (Figure 5C), SCF (Figure 5D), FGF (Figure 5E), or SDF (Figure 5F).

### Insulin/IGF/IRS mRNA expression

**FIGURE 6:**
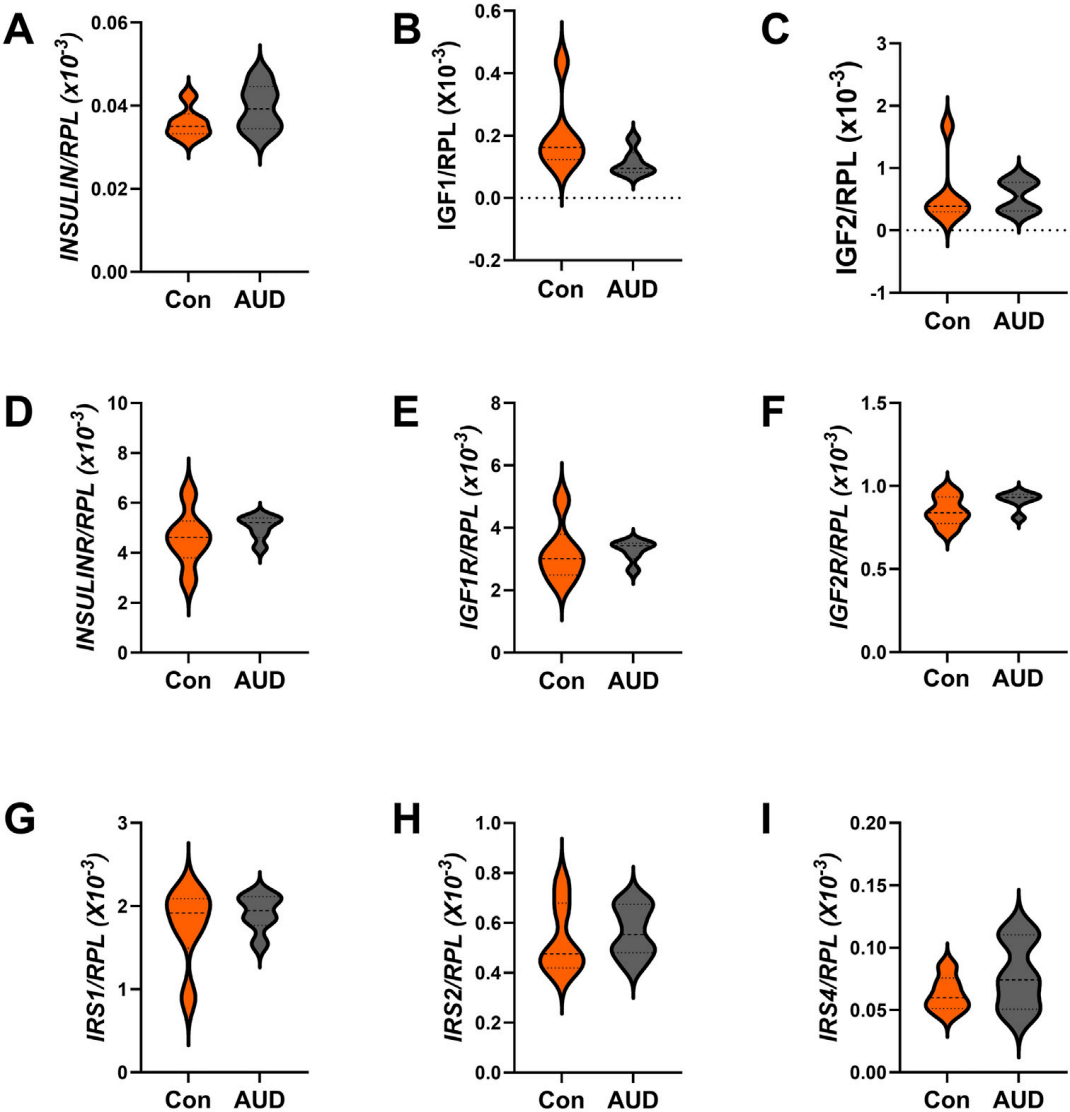
Effects of AUD on insulin/IGF/IRS pathway mRNA transcripts in the cerebellum. Relative mRNA transcript levels were measured with a custom multiplex magnetic bead-based RNA hybridization assay using 1 µg purified total RNA per sample (see Methods). Violin plots (median, quartiles, and range) display between-group differences in the levels of gene expression corresponding to **(A)** *INSULIN*, **(B)** *IGF1*, **(C)** *IGF2*, **(D)** *INSULINR*, **(E)** *IGF1R*, **(F)** *IGF2R*, **(G)** *IRS1*, **(H)** *IRS2*, and **(I)** *IRS4*, with results normalized to *RPL13a*. Data were analyzed by two-way ANOVA (Table 3) and *post hoc* multiple comparisons tests. Significant (p ≤ 0.05) differences are shown within the panels. See Supplementary Table S5 for abbreviations and full gene names, and the Heatmap in Figure 3B for comparative summary results.

For these studies, we compared the expression levels of insulin/IGF growth factors, their receptors, and IRS molecules using multiplex RNA hybridization assays of *INSULIN, IGF1, IGF2, INSULINR, IGF1R, IGF2R, IRS1, IRS2,* and *IRS4*. The rationale was that oligodendrocyte/myelin cellular functions are regulated by insulin/IGF pathways. The relative abundance of the mRNA transcripts was determined by calculating the ratios of target genes to RPL13a. The two-way ANOVA test revealed significant effects of insulin/IGF pathway mRNA transcript, but not AUD or AUD × insulin/IGF pathway mRNA transcript interactions (Table 3). Post hoc tests revealed that none of the AUD-associated changes in expression of *INSULIN/IGF/IRS* gene pathway molecules *were* statistically significant (Figure 6). Nonetheless, the corresponding heatmap showed that, except for *IGF1,* which was relatively reduced, AUD was associated with consistently higher levels of all *INSULIN/IGF/IRS* pathway mRNA transcripts (Figure 3B).

### Akt Pathway analysis

**FIGURE 7:**
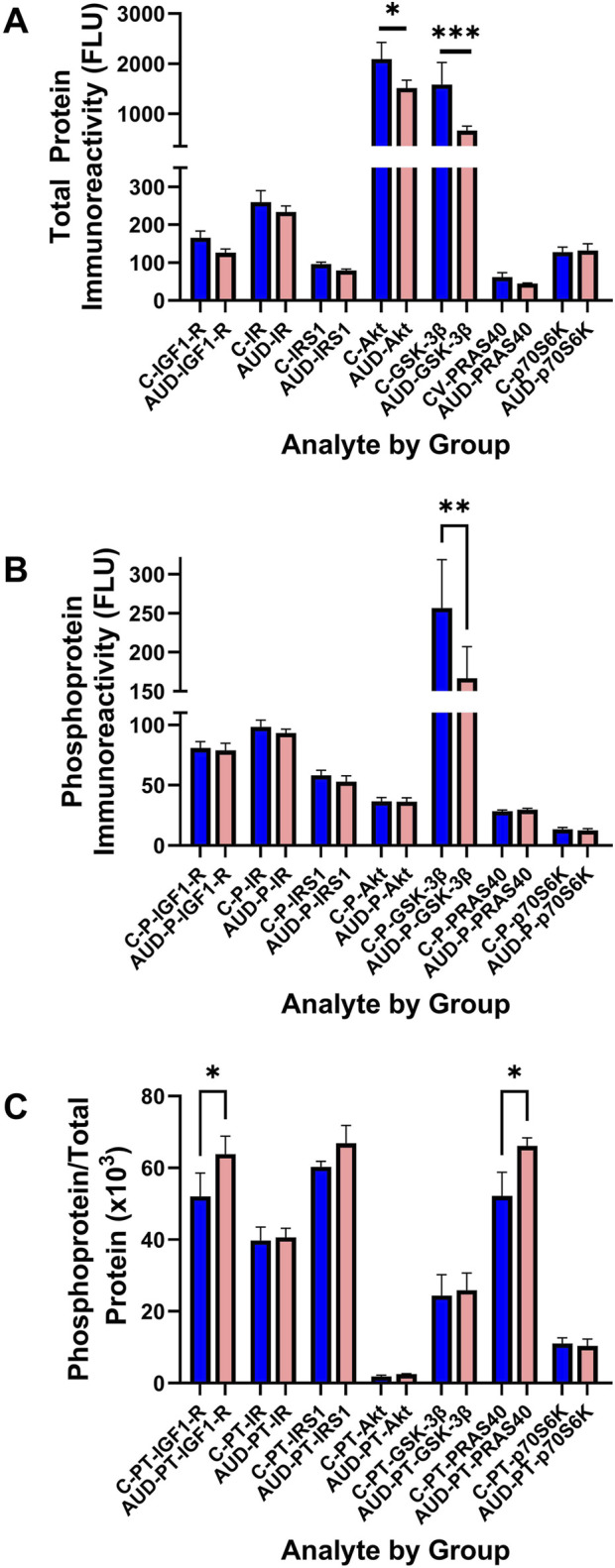
Human postmortem cerebellar vermis tissue homogenates from control (blue bars) and AUD (orange bars) donors were analyzed with commercial 7-plex **(A)** Akt and **(B)** phospho-Akt magnetic bead-based ELISA panels. The immunoreactivity results displayed correspond to fluorescent light units (FLU)/12.5 µg protein/well. **(C)** The relative levels of protein phosphorylation (PT) were calculated from the ratio of phosphorylated to total protein (see Supplementary Table S3 for abbreviation definitions). Graphs depict mean ± S.D. of Control (C-blue bars) versus AUD (orange bars) results. The data were analyzed using two-way ANOVA (see Table 2) with *post hoc* multiple comparison tests (*p < 0.05; **p < 0.01; ***p < 0.001). See Supplementary Table S3 for abbreviations.

To examine the effects of AUD on Insulin/IGF/IRS1 signaling through Akt pathways that lead to mTOR, we measured total and phosphorylated proteins using 7-plex bead-based ELISA platforms. In addition, to assess the effects on relative levels of phosphorylation, we calculated the ratios of phosphorylated to total protein immunoreactivity. Results were analyzed by two-way ANOVA with *post hoc* multiple comparison tests. The Two-way ANOVA tests detected significant AUD effects on the total protein and relative levels of phosphorylation, and significant Akt pathway biomarker effects on the total, phosphorylated, and relative phosphorylation levels of the signaling molecules. Additionally, a significant AUD × Akt pathway biomarker interaction was detected for total signaling protein expression (Table 2). The corresponding graphs display the within-group and between-group differences in total protein (Figure 7A), phosphoprotein (Figure 7B), and relative levels of protein phosphorylation (Figure 7C) with significant *post hoc* multiple comparisons test results. The main findings were significantly reduced Akt (Figure 7A), GSK-3β (Figure 7A), and pS-GSK-3β (Figure 7B), and increased pY/total-IGF1-R and pT/total-PRAS40 (Figure 7C) in AUD relative to control samples.

### Notch pathway mRNA studies

**FIGURE 8:**
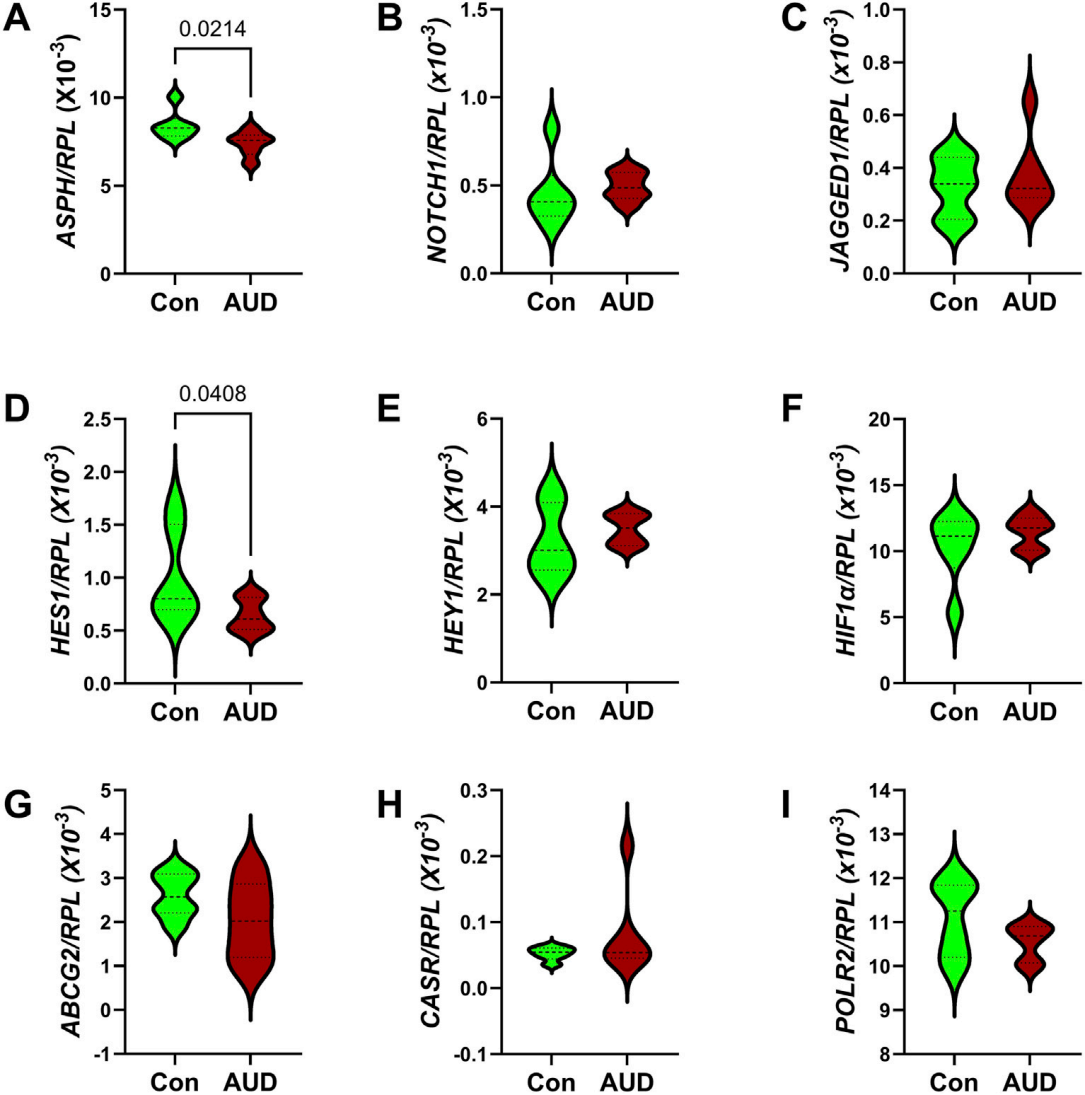
Effects of AUD on Notch pathway gene expression measured with a custom multiplex magnetic bead-based RNA hybridization panel using 1 µg purified total RNA per sample (see Methods). Violin plots (median, quartiles, and range) display between-group differences in the levels of gene expression corresponding to **(A)** *ASPH*, **(B)** *NOTCH1*, **(C)** *JAGGED1*, **(D)** *HES1*, **(E)** *HEY1*, **(F)** *HIF1α*, **(G)** *ABCG2*, **(H)** *CASR*, and **(I)** *POLR2*, with results normalized to *RPL13a*. Data were analyzed by two-way ANOVA (Table 3) and *post hoc* multiple comparisons tests. Significant (p ≤ 0.05) differences are shown within the panels. See Supplementary Table S5 for abbreviations and full gene names and Figure 3C for the Heatmap.

Notch pathway genes were evaluated because preclinical studies linked chronic heavy ethanol consumption and inhibition of ASPH and Notch pathway mRNA expression to white matter atrophy and cerebellar pathology [70]. Multiplex mRNA hybridization assays measured *ASPH, NOTCH1, JAGGED1, HES1, HEY1, HIF1α, ABCG2, CASR* and *POLR2a,* with results normalized to RPL13. Two-way ANOVA tests detected significant variance related to the Notch-related mRNA biomarkers, but not to AUD or the AUD × Notch-related mRNA biomarker interactive effects (Table 3). Violin plots with significant *post hoc* multiple comparison test results demonstrated AUD-associated reductions in ASPH (Figure 8A) and HES1 (Figure 8D), but none of the other mRNAs measured in the panel (Figures 8B,C,E–I). The corresponding heatmap, which includes the HPRT1 control gene, illustrates the within- and between-group differences in gene expression, along with the significant effects of AUD on ASPH and HES1 (Figure 3C).

## Discussion

The cerebellum is a major target of ARBD [6]. The damaging neurotoxic and degenerative effects of alcohol include neuronal loss and white matter atrophy, which account for motor impairments in AUD [3, 71]. Besides its critical roles in balance, bodily posture, and coordinated locomotion, the cerebellum has functional roles in motivation, reward learning, and social/emotional behaviors [72, 73]. Therefore, ARBD cerebellar-related impairments can be broad and include cognitive-behavioral dysfunctions [29]. Involvement of the cerebellar cortex is associated with loss of granule cells and Purkinje cells [3], whereas white matter damage is rooted in the myelin loss, oligodendrocyte dysfunction, neuroinflammation [3, 74–76], and astrocyte activation, i.e., gliosis with prominence of hypertrophic astrocytes.

Despite established evidence that white matter degeneration is a dominant feature of ARBD, its pathogenesis is poorly understood. The present work combines efforts to better understand the mechanisms of cerebellar and white matter degeneration caused by chronic alcohol misuse by characterizing oligodendrocyte-related molecular and biochemical pathologies in postmortem human AUD cerebella. The methodological approaches included measurements of immunoreactivity and mRNA transcripts, with the goal of obtaining complementary data to help confirm specific findings. However, not all results obtained by the two approaches were concordant, and several were opposite. Therefore, it is important to appreciate additional factors that differentially modulate protein and mRNA expression, including the potential for AUD-associated shifts in protein turnover, stability, and aggregation.

To investigate the effects of AUD on oligodendrocyte function, oligodendrocyte/myelin glycoprotein immunoreactivity and mRNA expression were measured by duplex ELISAs and multiplex RNA hybridization assays, which yielded complementary results in that elevated expression of CNPase and MOG was observed in the AUD samples using both approaches. However, the mRNA analysis was more sensitive and comprehensive for also demonstrating AUD-associated increases in KLK6, MBP, and PLP. The absence of AUD-associated reduction in GAPDH, a marker of insulin-stimulated metabolism [69], contrasts with experimental data showing that chronic brain insulin/IGF resistance, accompanied by oxidative stress and gliosis, contributes to altered myelin/oligodendrocyte glycoprotein gene expression and attendant oligodendrocyte dysfunction [42, 75, 77].

The increased expression of multiple oligodendrocyte/myelin glycoproteins in AUD brains is consistent with earlier findings in humans with AUD [45] and experimental ARBD [77], and likely reflects significant oligodendrocyte dysfunction. Previously, we demonstrated similar responses in the subacute stages of experimental ARBD, with broad inhibition of mRNA and immunoreactivity in the later stages of ARBD [78, 79]. None of the brains included in this study were from donors with advanced dementia, and therefore, their disease states were likely intermediate and comparable to the subacute chronic heavy exposure models. It is noteworthy that during development, chronic alcohol exposure instead causes severe WM pathology with prominent inhibition of oligodendrocyte/myelin glycoprotein expression [3, 39–41, 80], suggesting that the effects of ethanol differ for immature and mature oligodendrocytes. The increased levels of Kallikrein 6 (*KLK6*) serine protease could reflect responses in oligodendrocytes or activated microglia and astrocytes [81]. In active relapsing inflammatory demyelinating stages of multiple sclerosis, oligodendrocyte/myelin glycoproteins and Kallikreins were found to be reduced [82]. On the other hand, another study showed that *KLK6* and *KLK8* were upregulated in oligodendrocytes in response to CNS injury and myelin degradation, and that increased expression in microglia and astrocytes was functionally unrelated to myelination [81]. In addition, increased *KLK6* was observed in experimental autoimmune encephalomyelitis/MS and determined to represent a pathogenic mediator of inflammatory demyelination [83]. Inhibition of *KLK*6 expression in oligodendrocytes is associated with oligodendrocyte maturation and increased myelin thickness and volume [84], suggesting that its upregulation in AUD corresponds with failure of oligodendrocytes to fully mature, accounting for WM myelin loss.

The higher levels of oligodendrocyte glycoprotein expression in AUD suggest dysregulated responses related to chronic injury with altered myelin integrity, as demonstrated previously with lipidomic mass spectrometry [37] and possibly dysregulated oligodendrocyte function. To some degree, the abnormalities detected in human AUD brains mimic the findings in experimental models. The main difference is that in human cases, the upregulated expression of oligodendrocyte/myelin glycoproteins was broad, whereas in the experimental models, the immature oligodendrocyte/myelin glycoproteins were mainly increased, whereas the mature molecules were reduced by chronic ethanol exposure [77]. In essence, AUD with attendant ARBD significantly alters WM oligodendrocyte function, manifested by broad shifts in oligodendrocyte/myelin glycoprotein expression. Differential responses in human brains versus experimental animal models with ARBD could be linked to various human lifestyle co-factors such as smoking, nutrition [85], cannabis consumption [86], and variability in the rates, duration, consistency, and duration of alcohol misuse. Correspondingly, previous studies demonstrated reduced oligodendrocyte/myelin glycoprotein expression in WM of alcoholics with cirrhosis [87], which represents a more advanced stage of chronic alcohol-related organ damage than was evident in the AUD cases included in the present study.

In this study, the two-way ANOVA of the ELISA and demonstrated significant effects of glial/astrocyte biomarker indices, but the difference between AUD and control mean levels of GFAP immunoreactivity failed to reach statistical significance due to the high within-group variances. Similarly, the mean inter-group differences in GFAP mRNA expression failed to reach statistical significance (p = 0.0537). Although our ELISA results contrast with an earlier finding in which immunohistochemical staining showed elevated GFAP in AUD brains [88], the approach for measuring GFAP immunoreactivity by ELISA would have included soluble and not aggregated insoluble fractions that could contribute to immunostaining results. One potential consequence of astrocyte dysfunction is that it can contribute to neurobehavioral problems in AUD due to buildup of ethanol metabolites via increased aldehyde dehydrogenase 2 (ALDH2) expression, leading to acetate accumulation, GABA synthesis, and cerebellar dysfunction [89].

Although neuroinflammation is generally considered a driver of ARBD, a broad survey of proinflammatory cytokines, proinflammatory chemokines, and anti-inflammatory cytokines yielded largely negative results, together with significantly reduced expression of only one pro-inflammatory mediator, IL-16. IL-16 is a chemoattractant for CD4^+^ T cells and has a role in inflammatory demyelinating diseases [90]. Previous studies demonstrated upregulation of IL-16 in neurodegenerative diseases [91]. On balance, this study shows mainly no effect or reduced expression of pro-inflammatory mediators in cerebella of humans with AUD. Although our findings contrast with a number of studies linking increased neuroinflammatory responses to short-term, binge, or developmental alcohol exposures [92–96], they concur with other experimental model data showing that chronic ethanol consumption can suppress immune or cytokine-mediated responses [97, 98]. In essence, the findings herein do not provide supportive evidence that persistent chronic neuroinflammation is a prime mediator of WM ARBD.

Trophic factors mediate a broad range of growth-related functions in neurons, glia, and vascular elements. The AUD-associated significant reduction in HGF corresponds with neuronal loss in the cerebellar cortex in ARBD [3] since HGF has a key role in preventing neuronal death and promoting cell survival [99]. Given its pro-angiogenic and anti-inflammatory effects [100, 101] the failure to detect a significant AUD-associated reduction in SCF contrasts with data showing that low or moderate alcohol consumption adversely impacts neurogenesis [102, 103] or angiogenesis and brain perfusion [104, 105]. On the other hand, reduced expression of HGF likely has relevance to the blood-brain-barrier disruption that occurs in ARBD [100].

Impairments in CNS insulin and IGF signaling networks have been well documented in experimental models of ARBD [106, 107] but not in humans. The mRNA studies detected only modest AUD effects on insulin/IGF trophic factors and receptors, with the effects limited to a modest, non-significant increases in *INSULIN* and *IGF-2R*, and no changes in *IRS* mRNA transcript expression. Correspondingly, the AUD-associated effects insulin/IGF-Akt pathway molecules were restricted to reductions in Akt, GSK-3β, and S9-GSK-3β, suggesting possible inhibitory effects on PI3K-Akt signaling as reported in experimental models of chronic ethanol feeding [108–110]. However, the overall effects of AUD on insulin/IGF signaling networks in the cerebellum were modest compared with previous findings in experimental models [78, 98, 111] suggesting that other factors contribute to cerebellar degeneration in humans with AUD.

Notch pathway gene expression was measured because: 1) chronic ethanol exposure inhibits Notch signaling and ASPH expression in the brain [53, 55, 79]; 2) ASPH regulates Notch [59, 62]; 3) ASPH is expressed in WM [64] and its levels are reduced by alcohol exposure [54]; and 4) Notch is an important regulator of WM oligodendrocyte functions including myelin synthesis and maintenance [112, 113]. However, apart from cerebral autosomal dominant arteriopathy with subcortical infarcts and leukoencephalopathy (CADASIL), which is caused by Notch-3 mutations and associated with WM degeneration [63, 114], the effects of downregulated or impaired Notch signaling in human adult brains and neurodegeneration are largely unknown. Of note is that CADASIL, like WM ARBD, is associated with myelin loss and oligodendrocyte dysfunction [64]. The findings in the present study link AUD’s inhibitory effects on WM/oligodendrocyte integrity to a significant inhibition of *ASPH* and Notch pathway signaling via *HES1*. Therefore, AUD cerebellar pathology is mediated by downregulated expression of ASPH. The attendant impairment of Notch networks likely compromises oligodendrocyte functions needed to synthesize and maintain CNS WM myelin and thereby contributes to cognitive and motor dysfunctions associated with AUD/ARBD.

### Limitation of the study

Despite its novelty in the analysis of alcohol-related cerebellar white matter degeneration in humans, the study has limitations that should be addressed in future research. The subject numbers were modest (just 6 brains per group) due to limited samples available from cases with uncomplicated ARBD without co-existing problems caused by exposure to other drugs of abuse. The inclusion of males and not females is a weakness that could be explained by the male predominance of heavy drinkers, including in Australia.^1^ The study included only postmortem brains corresponding to ARBD endpoints. The research findings could be strengthened by examining brain tissue or other relevant specimens at earlier timepoints in ARBD. Finally, the inclusion of a subset of cases in which the participants ceased to drink heavily for a significant period would help assess abstinence-related reversibility of ARBD cerebellar WM pathology in humans.

## Data Availability

The raw data supporting the conclusions of this article will be made available by the authors, without undue reservation.

## Footnotes

1
https://aifs.gov.au/tentomen/insights-report/alcohol-use-among-australian-males
