# Supplementary material for: Molecular and biochemical pathologies in human alcohol-related cerebellar white matter degeneration
Source: Adv Drug Alcohol Res. 2025 Nov 3;5:15342. doi: 10.3389/adar.2025.15342 (PMC12620835; doi:10.3389/adar.2025.15342)
Supplement: Supplementary file 1 [file Supplementaryfile1.docx]

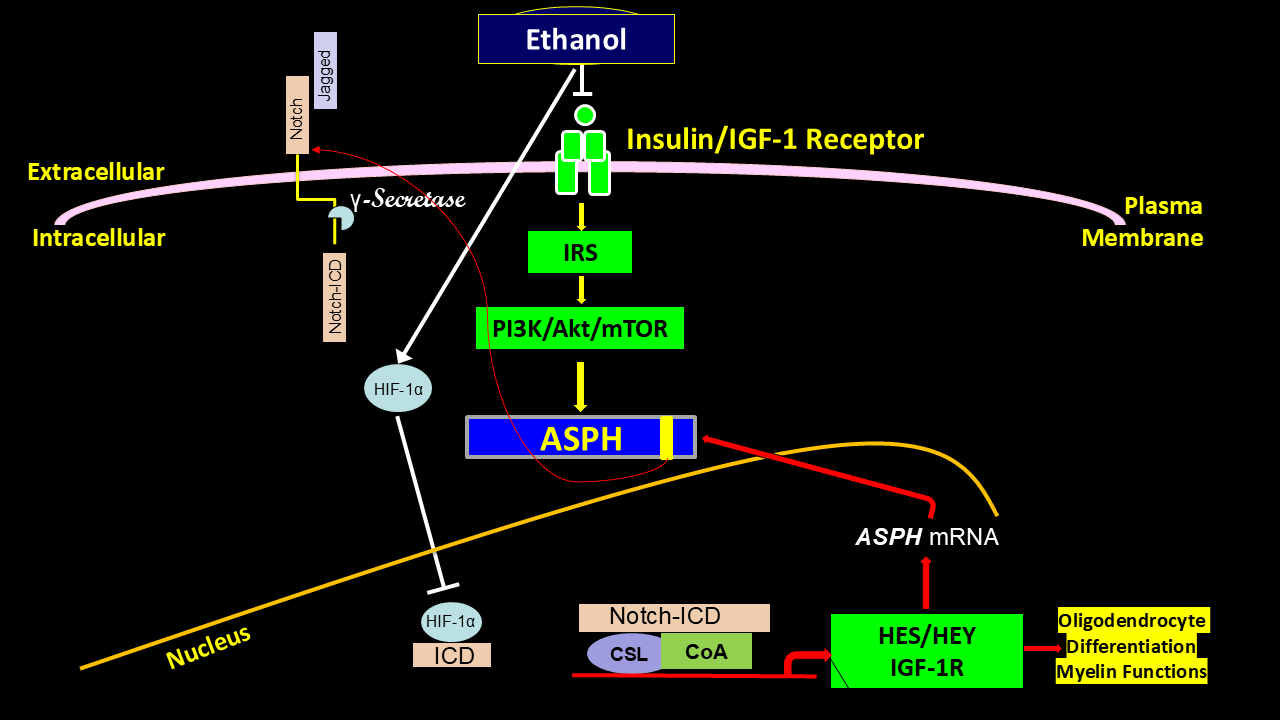
Supplementary Figure 1: Proposed Ethanol Effects on Crosstalk Between the Insulin/IGF and Notch Signaling Pathways
