# Supplementary material for: Molecular and biochemical pathologies in human alcohol-related cerebellar white matter degeneration
Source: Adv Drug Alcohol Res. 2025 Nov 3;5:15342. doi: 10.3389/adar.2025.15342 (PMC12620835; doi:10.3389/adar.2025.15342)
Supplement: Supplementary file 2 [file Table1.docx]

**Supplementary Table 1: Commercial Antibodies Used in Duplex ELISAs Including RRID and Validation**

| **Antibody Target abbreviation** | **Antibody Target (full name)** | **Source** | **Monoclonal/**  **Polyclonal** | **Stock (mg/mL)** | **Final Dilution (µg/mL)** | **Commercial Source** | **Reference*** | **RRID** | **Validation from Manufacturer** |
| --- | --- | --- | --- | --- | --- | --- | --- | --- | --- |
| CNPase (11-5B) | 2',3'-cyclic nucleotide 3' phosphodiesterase | Mouse | Monoclonal | 1.0 | 2.0 | Abcam, Boston, MA | ab6319 | AB_2082593 | western blot, ELISA, immunoprecipitation, immunohistochemistry, immunocytochemistry |
| GALC protein; GC-globulin) | Group-specific component Vitamin D Binding | Rabbit | Polyclonal | 1.0 | 2.0 | Abcam, Boston, MA | ab83752 | AB_2108528 | ELISA; Western Blot; WB |
| MAG1 | Myelin-Associated Glycoprotein 1 | Mouse | Monoclonal | 0.5 | 0.25 | Abcam, Boston, MA | ab89780 | AB_2042411 | ELISA; Western Blot; ELISA, Western Blot |
| MOG | Myelin Oligodendrocyte Glycoprotein | Rabbit | Polyclonal | 1.0 | 2.0 | Abcam, Boston, MA | ab32760 | AB_2145529 | western blot, immunohistochemistry |
| MBP | Myelin basic protein | Rabbit | Polyclonal | 1.0 | 2.0 | MilliporeSigma, Burlington, MA | M3821 | AB_1841021 | Uccelli NA, Codagnone MG, Traetta ME, Levanovich N, Rosato Siri MV, Urrutia L, Falasco G, Vázquez S, Pasquini JM, Reinés AG. Neurobiological substrates underlying corpus callosum hypoconnectivity and brain metabolic patterns in the valproic acid rat model of autism spectrum disorder. J Neurochem. 2021 Oct;159(1):128-144. doi: 10.1111/jnc.15444. Epub 2021 Jun 28. PMID: 34081798. |
| PLP | Proteolipid Protein 1 | Rabbit | Polyclonal | Serum | 1:2000 | Abcam, Boston, MA | ab28486 | AB_776593 | Immunocytochemistry; Immunohistochemistry; Western Blot; Immunocytochemistry, Immunocytochemistry/Immunofluorescence, Immunohistochemistry-Fr, Immunohistochemistry-P, Western Blot |
| PDGFRA | Platelet-derived growth factor receptor, alpha polypeptide | Rabbit | Polyclonal | 1.0 | 1.0 | Abcam, Boston, MA | ab61219 | AB_2162341 | Immunofluorescence; Western Blot; Immunocytochemistry; Immunohistochemistry - fixed; Immunohistochemistry; ELISA; ELISA, ICC/IF, IHC-P, WB |
| Nestin | Nestin | Rabbit | Polyclonal | Serum | 1:2000 | Abcam, Boston, MA | ab27952 | AB_776698 | Immunofluorescence; Western Blot; Immunocytochemistry/Immunofluorescence, Immunofluorescence, Western Blot |
| Vimentin | Vimentin | Mouse | Monoclonal | 1.0 | 2.5 | Abcam, Boston, MA | ab8978 | AB_306907 | Flow Cyt, ICC, ICC/IF, IHC (PFA fixed), IHC-Fr, IHC-P, IP, WB; Flow Cytometry; Immunofluorescence; Immunohistochemistry; Western Blot; Immunohistochemistry - frozen; Immunoprecipitation; Immunocytochemistry; Immunohistochemistry - fixed |
| GFAP | Glial Fibrillary Acidic Protein | Goat | Polyclonal | 0.5 | 0.5 | Abcam, Boston, MA | ab53554 | AB_880202 | ELISA, ICC/IF, IHC-Fr, IHC-FrFl, sELISA, WB; ELISA; Immunohistochemistry - frozen; Immunohistochemistry; Western Blot; Immunofluorescence; Immunocytochemistry |
| RPLPO | Large acidic ribosomal protein | Mouse | Monoclonal | 1.0 | 0.1 | Proteintech, Chicago, IL | AG1829 | AB_2254064 | <http://antibodyregistry.org/AB_2254064>;  Originating manufacturer of this product. Applications: WB, IP, IHC, IF, ELISA |

*RRID= Research Resource Identifier
