# Supplementary material for: Molecular and biochemical pathologies in human alcohol-related cerebellar white matter degeneration
Source: Adv Drug Alcohol Res. 2025 Nov 3;5:15342. doi: 10.3389/adar.2025.15342 (PMC12620835; doi:10.3389/adar.2025.15342)
Supplement: Supplementary file 4 [file Table5.docx]

| Gene Code | Gene Name |
| --- | --- |
| **Insulin/IGF/IRS Pathway** | |
| *INS* | Insulin |
| *IGF1* | Insulin-Like Growth Factor 1 |
| *IGF2* | Insulin-Like Growth Factor 2 |
| *INSR* | Insulin Receptor |
| *IGF1R* | Insulin-Like Growth Factor 1 Receptor |
| *IGF2R* | Insulin-Like Growth Factor 2 Receptor |
| *IRS1* | Insulin Receptor Substrate, Type 1 |
| *IRS2* | Insulin Receptor Substrate, Type 2 |
| *IRS4* | Insulin Receptor Substrate, Type 4 |
| **Notch Pathway** | |
| *ASPH* | Aspartyl-Asparaginyl-β-Hydroxylase |
| *NOTCH1* | Notch 1 |
| *JAG1* | Jagged 1 |
| *HES1* | Hairy and enhancer of split-1 |
| *HEY1* | HES-related family bHLH transcription factor |
| *HIF1α* | Hypoxia-Inducible Factor 1-alpha |
|  |  |
| *ABCG2* | ATP-binding cassette family gene 2 |
| *CASR* | Calcium-Sensing Receptor |
| *HPRT1* | Hypoxanthine Phosphoribosyltransferase 1 |
| *POLR2a* | RNA Polymerase II Subunit A |
| **Control Gene** | |
| *RPL13a* | Ribosomal Protein L13a |

**Supplementary Table 5: Quantigene 2.0 Insulin/IGF/IRS-Notch Pathway**
