# Supplementary material for: Molecular and biochemical pathologies in human alcohol-related cerebellar white matter degeneration
Source: Adv Drug Alcohol Res. 2025 Nov 3;5:15342. doi: 10.3389/adar.2025.15342 (PMC12620835; doi:10.3389/adar.2025.15342)
Supplement: Supplementary file 5 [file Table3.docx]

| **Insulin/IGF-Akt Pathway Molecules** | **Protein** | **Phosphoprotein** |
| --- | --- | --- |
| Insulin Receptor | Insulin-R | ^pYpY1162/1163^-Insulin R |
| Insulin-Like Growth Factor Receptor Type 1 | IGF1-R | ^pYpY1135/1136^-IGF1-R |
| Insulin Receptor Substrate, Type 1 | IRS1 | ^pS636^-IRS1 |
| Akt (Protein Kinase B) | Akt | ^pS473^-Akt |
| Glycogen Synthase Kinase 3β | GSK-3β | ^pS9^-GSK3β |
| p70 Ribosomal S6 kinase | P70S6K | ^pT412^-p70S6K |
| Ribosomal Protein S6 | RPS6 | ^pS235/S236^-RPS6 |

**Supplementary Table 3: Total and Phosphoprotein Akt Targets in the ELISA Panels**

Commercial magnetic bead-based total and phosphoprotein 7-plex Akt ELISA panels used to measure insulin/IGF signaling through Akt pathways.
