# Supplementary material for: Molecular and biochemical pathologies in human alcohol-related cerebellar white matter degeneration
Source: Adv Drug Alcohol Res. 2025 Nov 3;5:15342. doi: 10.3389/adar.2025.15342 (PMC12620835; doi:10.3389/adar.2025.15342)
Supplement: Supplementary file 6 [file Table4.docx]

| **Abbreviation** | **Full Name** | **Gene Names** |
| --- | --- | --- |
| ***CNP*** | 2',3'-cyclic nucleotide 3' phosphodiesterase | *CNPase, EC, CNP1* |
| ***CSPG4*** | Chondroitin Sulfate Proteoglycan 4 | *MCSP;* Melanoma-Associated Chondroitin Sulfate |
| ***GFAP*** | Glial Fibrillary Acidic Protein | *GFAP;* Intermediate Filament Protein |
| ***KLK6*** | Kallikrein-related peptidase 6 | *KLK6* |
| ***KLK8*** | Kallikrein-related peptidase 8/ neuropsin | *KLK8* |
| ***MBP*** | Myelin Basic Protein | *MBP; Myelin A1 Protein* |
| ***MOG*** | Myelin Oligodendrocyte Glycoprotein | *MOGIG2* |
| ***PLP1*** | Proteolipid Protein 1 | *PLP, SPG2* |
| ***GAPDH*** | Glyceraldehyde-3-phosphate dehydrogenase | *GAPD; G3PD* |
| ***HPRT1*** | Hypoxanthine phosphoribosyltransferase 1 | *HPRT* |
| ***RPL13A*** | Ribosomal Protein L13a | *L13A; UL13* |

**Supplementary Table 4: Quantigene 2.0 Glial Panel**
